# Capacity assessment of the health laboratory system in two resource-limited provinces in China

**DOI:** 10.1186/s12889-019-6777-2

**Published:** 2019-05-10

**Authors:** Bo Liu, Fang Ma, Jeanette J. Rainey, Xin Liu, John Klena, Xiaoyu Liu, Biao Kan, Meiying Yan, Dingming Wang, Yan Zhou, Guangpeng Tang, Mingliu Wang, Chihong Zhao

**Affiliations:** 1 Office of Laboratory Management, Chinese Center for Disease Control and Prevention, Room 335, 155 Changbai Road, Changping District, Beijing, 102206 People’s Republic of China; 2 Emerging and Infectious Disease Program, Centers for Disease Control and Prevention, Beijing, 100600 China; 3 Division of Global Health Protection, Centers for Disease Control and Prevention, Atlanta, GA 30329-4027 USA; 4 National Institute for Communicable Disease Control and Prevention (ICDC), Chinese Center for Disease Control and Prevention, Beijing, 102206 China; 5 Guizhou Center for Disease Control and Prevention, Guizhou Province, 550004 China; 6 Guangxi Center for Disease Control and Prevention, Guangxi Province, 530028 China

**Keywords:** International health regulations, Laboratory assessment, Public health laboratory, Clinical laboratory, Resource-limited, Guangxi, Guizhou, China

## Abstract

**Background:**

Strong laboratory capacity is essential for detecting and responding to emerging and re-emerging global health threats. We conducted a quantitative laboratory assessment during 2014–2015 in two resource-limited provinces in southern China, Guangxi and Guizhou in order to guide strategies for strengthening core capacities as required by the International Health Regulations (IHR 2005).

**Methods:**

We selected 28 public health and clinical laboratories from the provincial, prefecture and county levels through a quasi-random sampling approach. The 11-module World Health Organization (WHO) laboratory assessment tool was adapted to the local context in China. At each laboratory, modules were scored 0–100% through a combination of paper surveys, in-person interviews, and visual inspections. We defined module scores as strong (> = 85%), good (70–84%), weak (50–69%), and very weak (< 50%). We estimated overall capacity and compared module scores across the provincial, prefecture, and county levels.

**Results:**

Overall, laboratories in both provinces received strong or good scores for 10 of the 11 modules. These findings were primarily driven by strong and good scores from the two provincial level laboratories; prefecture and county laboratories were strong or good for only 8 and 6 modules, respectively. County laboratories received weak scores in 4 modules. The module, ‘Public Health Functions’ (e.g., surveillance and reporting practices) lagged far behind all other modules (mean score = 46%) across all three administrative levels. Findings across the two provinces were similar.

**Conclusions:**

Laboratories in Guangxi and Guizhou are generally performing well in laboratory capacity as required by IHR. However, we recommend targeted interventions particularly for county-level laboratories, where we identified a number of gaps. Given the importance of surveillance and reporting, addressing gaps in public health functions is likely to have the greatest positive impact for IHR requirements. The quantitative WHO laboratory assessment tool was useful in identifying both comparative strengths and weaknesses. However, prior to future assessments, the tool may need to be aligned with the new WHO IHR monitoring and evaluation framework.

**Electronic supplementary material:**

The online version of this article (10.1186/s12889-019-6777-2) contains supplementary material, which is available to authorized users.

## Background

The International Health Regulations (IHR, 2005) published by the World Health Organization (WHO) describe the minimum core capacities required to detect, assess, report, and respond to public health emergencies of international concern [1]. China follows the IHR in responding to common threats such as seasonal Influenza A as well as transmission of novel infections such as Severe Acute Respiratory Disease Syndrome (SARS) [2]. Strong laboratory capacity is essential for detecting and responding to public health threats as outlined in the Regulations.

In China, front-line detection and response responsibilities rely on the provincial-, prefecture-, and county-level Centers for Disease Control. At each administrative level, public health laboratories collaborate with hospital-based clinical laboratories to investigate suspected cases of emerging and re-emerging infections. County- and prefecture-level laboratories conduct field-based epidemiological investigations and perform initial specimen collection and testing, and provincial-level laboratories provide molecular testing and sub-typing for further identification purposes and coordinate surveillance activities.

Maintaining laboratory capacity across each administrative level and ensuring collaboration between the public health and clinical laboratories are key for full IHR adherence. Since maintaining capacity and ensuring collaboration could be more challenging in resource-limited areas, we conducted a systematic laboratory assessment in two resource-limited provinces in southern China, Guangxi and Guizhou, using the Laboratory Assessment Tool (LAT) developed by WHO. To the best of our knowledge, this is the first application and publication of quantitative scores for assessing laboratory capacity in multiple provinces of China. Findings from this assessment will be used to generate data-drive evidence on current laboratory capacity and need for specific interventions in Guangxi and Guizhou Provinces.

## Methods

### Assessment provinces and laboratory selection

We conducted this assessment in two resource-limited provinces of China, Guizhou and Guangxi. In 2014 the populations of Guangxi and Guizhou were 47,540,000 and 35,080,000, respectively. The average annual Gross Domestic Product (GDP) for these provinces is approximately $194.7 billion [3], substantially lower than the national average of $295.9 billion [4]. The provinces are administratively divided into prefectures and counties; counties are further divided into townships. Outbreaks of *Salmonella* typhi and paratyphi are common in Guangxi and Guizhou [5, 6]. Both provinces have detected Avian Influenza A H7N9 [7, 8].

We used a stratified random sampling approach to select two prefectures in Guizhou (22.2% of all prefectures) and two in Guangxi (14.3% of all prefectures). Then from each of these four prefectures, we randomly selected two counties (26.7 and 19.0% of the counties in the two selected prefectures in Guizhou and Guangxi, respectively). At each administrative level, laboratories from both public health and clinical sectors were selected. The selection approach resulted in a total of 14 laboratories for each province (provincial level [*n* = 2 laboratories], prefecture [*n* = 4], and county [*n* = 8]), including 7 hospital-based clinical laboratories. Public laboratories (PHLs) and clinical laboratories (CLs) are managed by separate agencies at each administrative level.

### Assessment tool

The WHO LAT is a generic document containing a number of modules and indicators that can be adapted by public health officials to evaluate specific core laboratory capacities and capabilities at the national or local levels. For this assessment, we selected 11 modules from the WHO LAT (Table 1). These modules were adapted to the local context (i.e., modified wording to improve interpretation), translated into Chinese and pilot-tested prior to initiating the assessment. Each of the 11 modules included multiple indicators (ranging from 3 to 10 per module), quantitatively scored between 0 and 100% [9]. The module score was calculated as the average across the module’s indicators. We generated the overall laboratory assessment score as the average of the 11 module scores. Standard deviations were included to measure the spread or distribution around each calculated average.

**Table 1:** Assessment modules included in the adapted WHO Laboratory Assessment Tool used in Guangxi and Guizhou Provinces, China, 2014

Modules	Laboratory Capacities
Organization & management	Internal & external communication, budget, licensing/supervision/accreditation
Documents	Document control system, quality procedures, biosafety documents
Specimen collection, handling & transport	Specimen collection, handling, referral/transport
Data & information management	Test results and reports, data analysis and statistics, data security & confidentiality, it and laboratory information management system (LIMS)
Consumables & reagents	Procurement, inventory and storage, use, expired reagents
Equipment	Equipment inventory, maintenance, calibration and monitoring
Laboratory testing performance	All relevant tests performed, concerning bacteriology, virology, parasitology and food
Facilities	Infrastructure, work conditions
Human resources	Staff number, qualifications, continuous education
Biorisk management	Biorisk management policy, biorisk assessment and control, implementation and operation
Public health functions	Surveillance and response, specimens and reporting for public health purposes

### Data collection and analysis

We obtained initial information through assessment surveys mailed to the laboratory manager at each selected laboratory. Two teams of two members each conducted on-site visits to verify these self-administered surveys as well as to conduct laboratory inspections. Team members received training on the use of the assessment tool and interview techniques by the China CDC staff. The assessments were conducted from July to November 2014 in both provinces. Verification and inspection data were documented on standardized paper forms.

We double-entered all survey and verification data into an Excel database and reconciled identified inconsistencies and missing data prior to conducting the descriptive analysis. We compared and contrasted assessment findings across the three administrative levels as well as between public health laboratories (PHLs) and clinical laboratories (CLs). We categorized module scores as strong (> = 85%), good (70–84%), weak (50–69%) and very weak (< 50%). We examined and described the individual indicators for modules where the scores for any administrative level was < 70%. Data were entered into SPSS (version 16.0, New York, USA) for analysis, assuming quasi-random sampling (i.e., results were not adjusted for varying selection probabilities within and across the two provinces).

No personal identifying information from patients or specimens was obtained during the assessment. China CDC and Guangxi and Guizhou Provincial CDCs approved the assessment as non-research. The assessment was also determined to be non-research by the Human Subjects Research Determination Process at the United States Centers for Disease Control and Prevention and therefore exempt from IRB review.

## Results

### Overall assessment scores

On average, the laboratories selected for this assessment received an overall good (75.7%) score across the 11 modules (Table 2). Provincial laboratories received the highest overall score (79.6%) followed by prefecture (77.8%) and county (73.7%) laboratories. Among the 11 modules, laboratories were overall strong (≥85%) in ‘Equipment’ and ‘Specimen Collection’ and good (≥ 70%) in ‘Organization and Management’, ‘Documents’, ‘Specimen Handling and Transport’, ‘Data and Information Management’, ‘Consumables and Reagents’, ‘Laboratory Testing Performance’, ‘Facilities’, ‘Human Resources’ and ‘Biorisk Management’. Laboratories were weak or very weak (≤ 50%) in ‘Public Health Functions’. These findings were generally similar across the two provinces.

**Table 2:** Aggregated performance scores on 11 assessment modules for 28 laboratories, categorized by administrative level (provincial, prefecture, and county) and laboratory sector (Public Health - PH, and Clinical - CL), in Guangxi and Guizhou Provinces, China, 2014

Assessment modules	Average (%)	Provincial (%)			Prefecture (%)			County (%)		
		All	PH	CL	All	PH	CL	All	PH	CL
Average	75.7	79.6 (13.5)	81.5 (15.3)	77.6 (17.3)	77.8 (7.6)	75.9 (5.3)	79.8 (9.9)	73.7 (14.6)	72.8 (14.8)	74.6 (15.5)
Organization & management	71.4	77.6 (12.4)	74.3 (18.6)	80.9 (8.4)	74.7 (14.5)	66.1 (5.8)	83.3 (16.1)	68.1 (18.3)	67.5 (25.2)	68.8 (8.9)
Documents	75.4	87.7 (20)	97.7 (3.2)	77.6 (27.9)	83.6 (15.3)	88.3 (9.4)	78.9 (19.9)	68.2 (27.8)	74.1 (30.5)	62.3 (25.4)
Specimen collection, handling & transport	85.3	80.4 (11.2)	77.4 (15.2)	83.4 (10.6)	89.1 (6.2)	87.1 (7.4)	91 (5.1)	84.6 (14)	87 (10.7)	82.3 (17.1)
Data & information management	81.6	75.8 (17)	71.3 (24.7)	80.3 (13.3)	82.6 (12.3)	82.2 (16.2)	83 (9.6)	82.6 (14.6)	85.1 (14.9)	80.1 (14.8)
Consumables & reagents	83.3	85.5 (12.4)	85.4 (15.2)	85.7 (15.2)	83.8 (9.6)	79.6 (7.1)	88.1 (10.8)	82.5 (15.7)	80.3 (12.2)	84.6 (19.2)
Equipment	87.6	84.5 (16.3)	75 (20.5)	94 (4.8)	90.9 (11)	89.5 (13.8)	92.3 (9.4)	86.8 (18.5)	88.6 (14.1)	84.9 (23)
Laboratory testing performance	71.3	70.7 (41.9)	87.2 (2.7)	54.3 (64.7)	68.4 (22.6)	64.9 (27.9)	71.8 (19.5)	72.9 (23.4)	73.8 (24.9)	72 (23.6)
Facilities	71.5	78.4 (22)	70.5 (34.7)	86.4 (0)	75.1 (21.1)	63.6 (24.5)	86.5 (9.7)	67.9 (18.9)	69 (19.6)	66.8 (19.6)
Human resources	79.5	88.8 (6.3)	85 (7.1)	92.5 (3.5)	80 (16.3)	68.8 (13.1)	91.3 (10.3)	76.9 (16.2)	71.9 (14.6)	81.9 (17.1)
Biorisk management	75.9	89.6 (10.9)	87.1 (14.4)	92.1 (11.2)	81.1 (18.8)	72.9 (23.2)	89.3 (10.6)	69.9 (29.9)	57.5 (32.8)	82.3 (22)
Public health functions	47.6	56 (40.7)	85.1 (11.6)	26.9 (38.1)	46.8 (31.8)	71.7 (17.7)	21.9 (19.8)	45.7 (27.8)	46.5 (26.5)	44.6 (32)

Module scores are defined as strong (> = 85%), good (70–84%), weak (50–69%), and very weak (< 50%)

### Assessment scores by administrative level

**Fig. 1:**
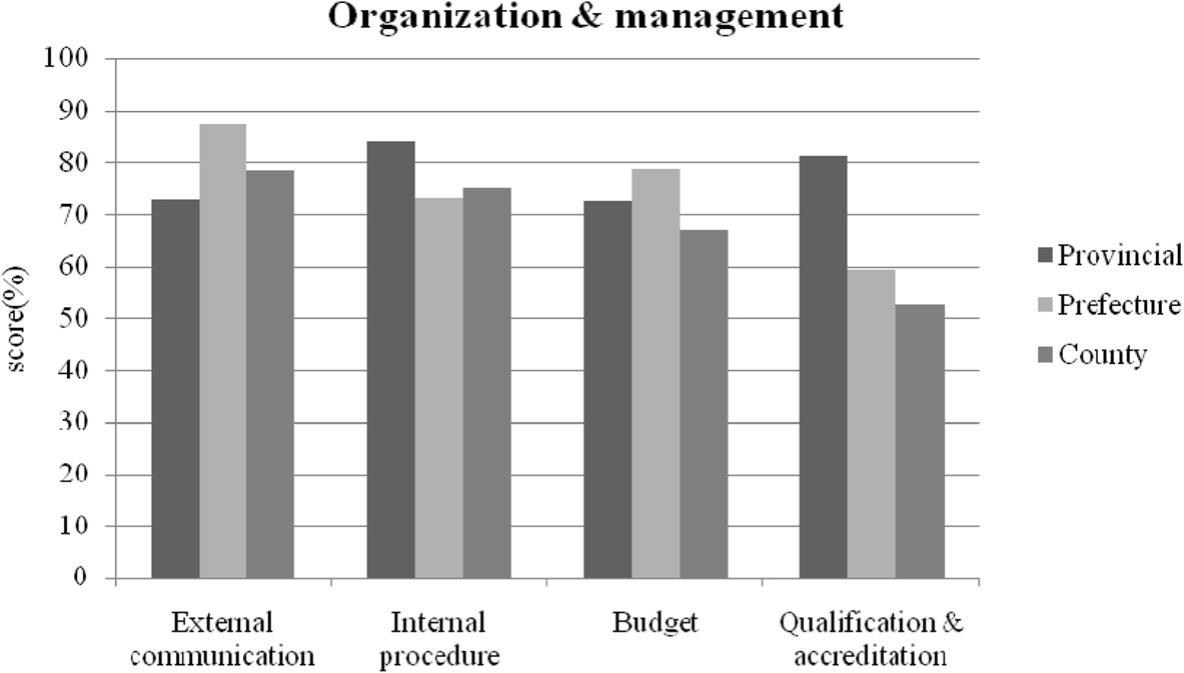
Performance scores of the ‘Organization and Management’ module for the 28 laboratories by administrative levels (county, prefecture and province), Guangxi and Guizhou Provinces, China, 2014. Note: The average module score at the provincial-level = 77.6%, prefectural-level = 74.7% and, county-level = 68.1%

**Fig. 2:**
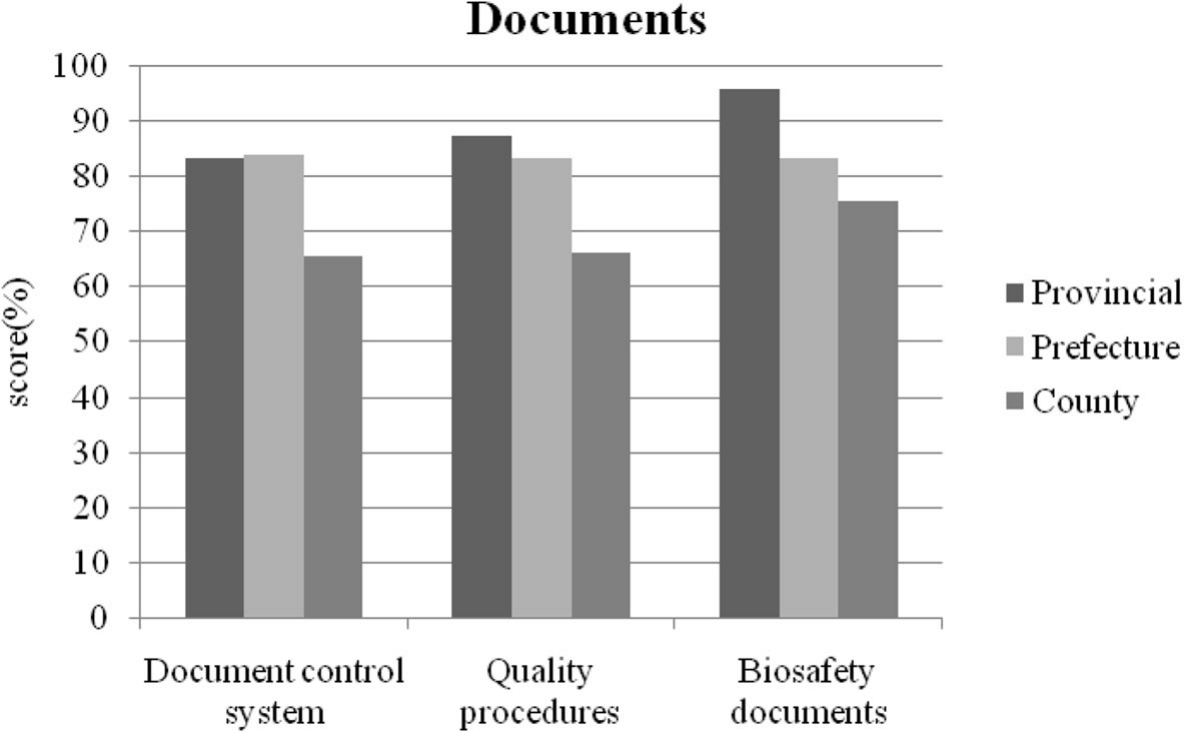
Performance scores of the ‘Documents’ module for the 28 laboratories by administrative levels (county, prefecture and province), Guangxi and Guizhou Provinces, China, 2014. Note: The average module score at the provincial-level = 87.7%, prefectural-level = 83.6%, and county-level = 68.2%

**Fig. 3:**
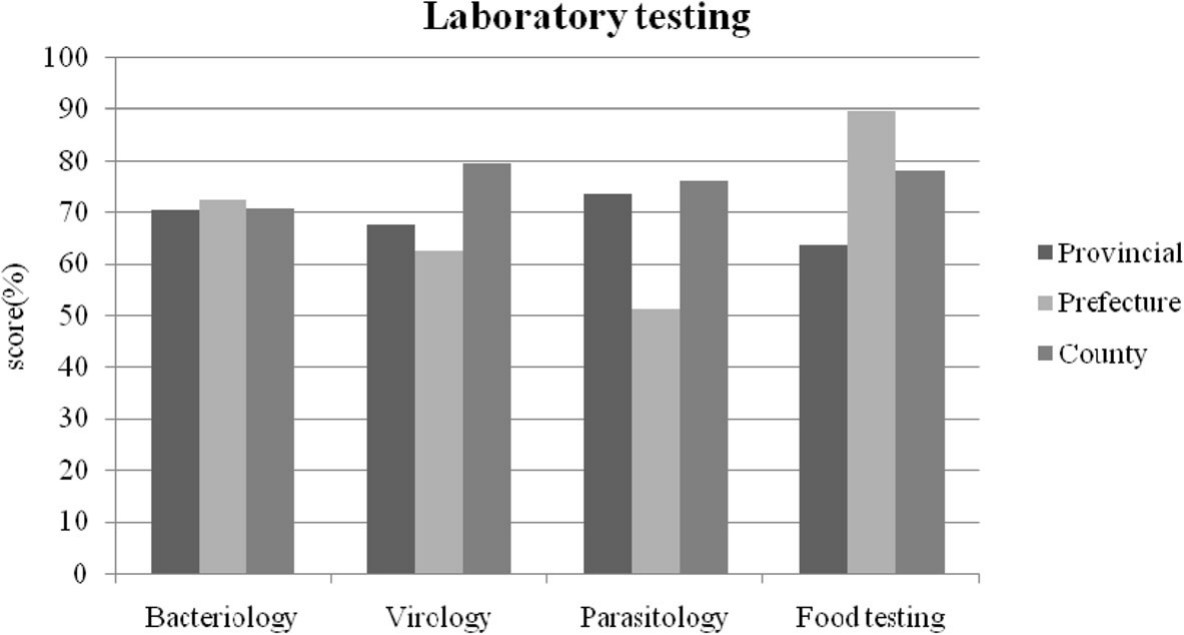
Performance scores of the ‘Laboratory Testing’ module for the 28 laboratories by administrative levels (county, prefecture and province), Guangxi and Guizhou Provinces, China, 2014. Note: The average module score at the provincial-level = 70.7%, prefectural-level = 68.4%, and county-level = 72.9%

**Fig. 4:**
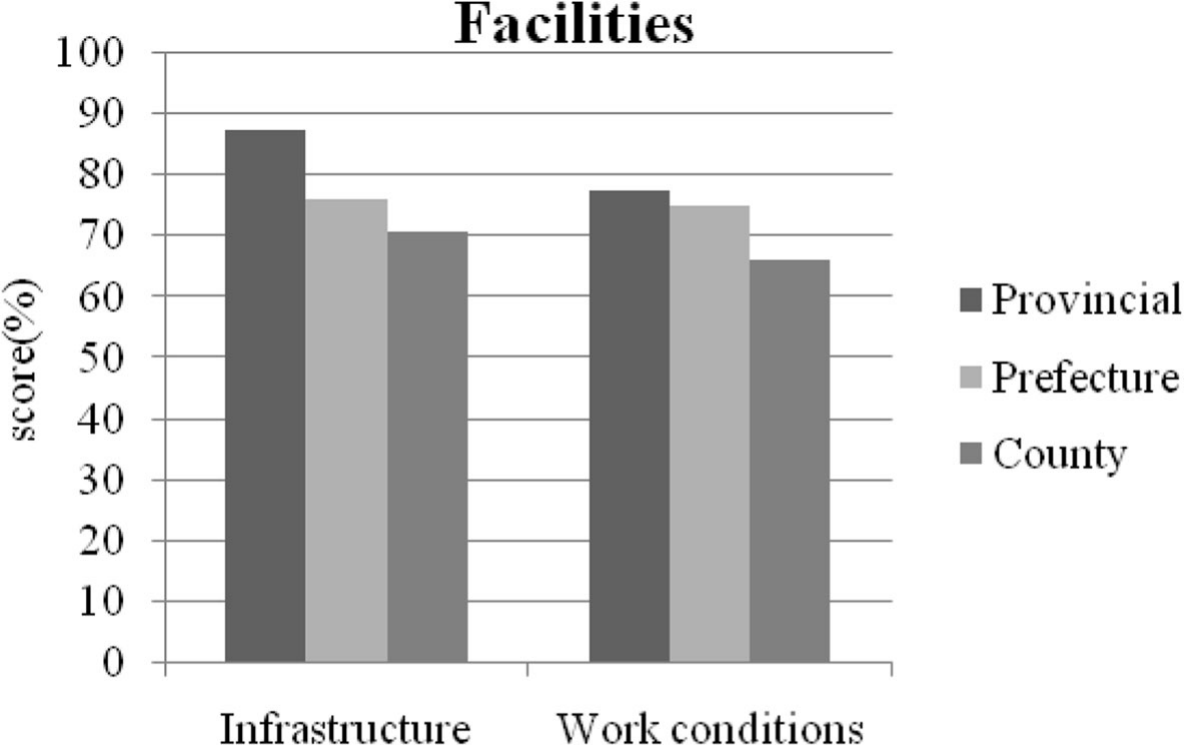
Performance scores of the ‘Facilities’ module for the 28 laboratories by administrative levels (county, prefecture and province), Guangxi and Guizhou Provinces, China, 2014. Note: The average module score at the provincial-level = 78.4%, prefectural-level = 75.1%, and county-level = 67.9%

**Fig. 5:**
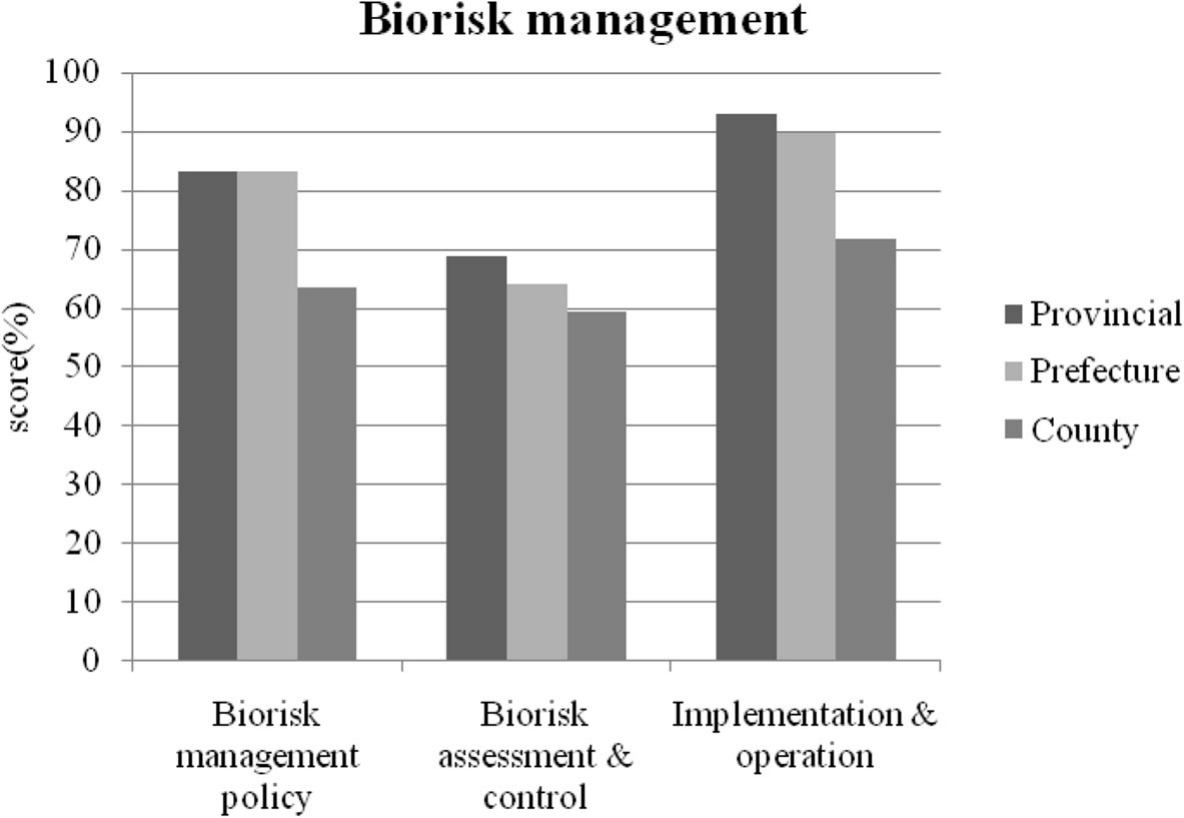
Performance scores of the ‘Biorisk Management’ module for the 28 laboratories by administrative levels (county, prefecture and province), Guangxi and Guizhou Provinces, China, 2014. Note: The average module score at the provincial-level = 89.6%, prefectural-level = 81.1%, and county-level = 69.9%

**Fig. 6:**
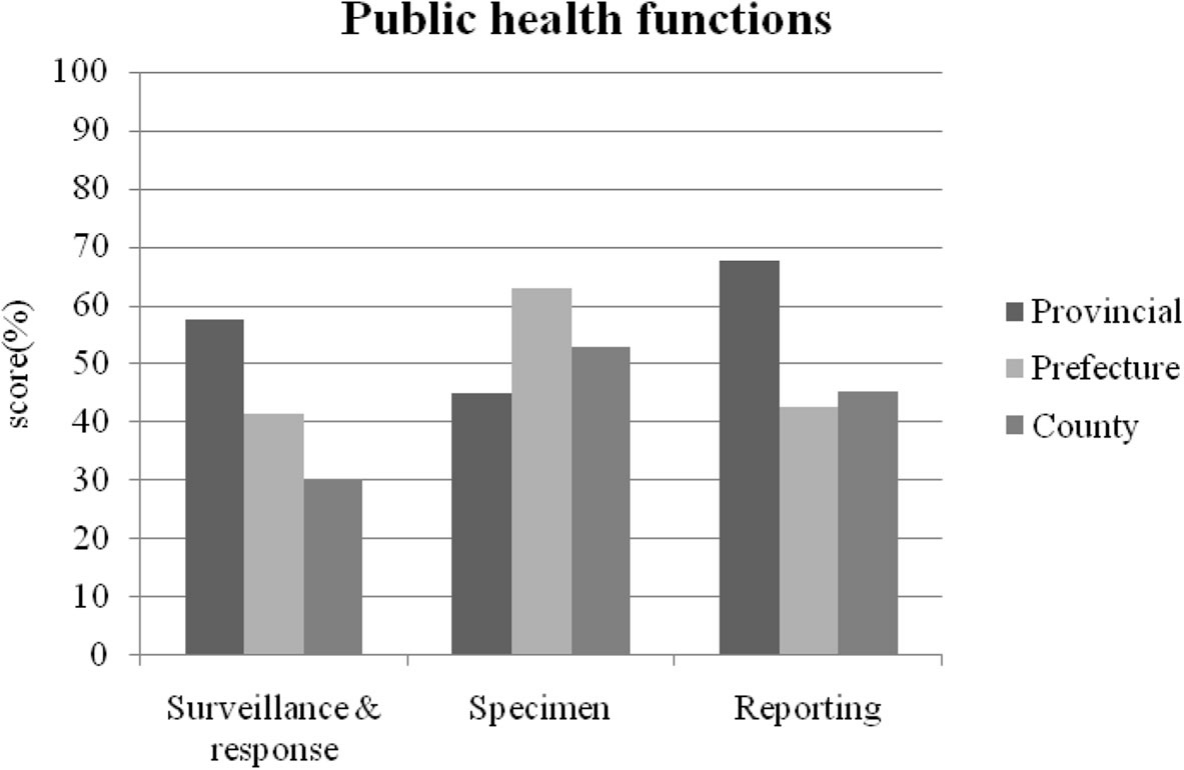
Performance scores of the ‘Public Health Functions’ module for the 28 laboratories by administrative levels (county, prefecture and province), Guangxi and Guizhou Provinces, China, 2014. Note: The average module score at the provincial-level = 56%, prefectural-level = 46.8%, and county-level = 45.7%

At least one administrative level scored weak, or < 70%, for the following six modules, ‘Organization and Management’, ‘Documents’, ‘Laboratory Testing Performance’, ‘Facilities’, ‘Biorisk Management’ and ‘Public Health Functions’. This finding was primarily driven by weak county-level scores. The exception was for ‘Laboratory Testing Performance’ where only prefecture level laboratories scored < 70%. To better understand the ‘causes’ of these weak scores, the individual indicators for these six modules are described below (Figs. 1, 2, 3, 4, 5, 6). We describe differences in module scores between public health and clinical laboratories separately below.

#### Organization and management

The ‘Organization and Management’ module scores reflect the average across five separate indicators (Fig. 1). County-level laboratories scored weak on the ‘Budget’ (51%) and ‘Qualification and Accreditation’ (68%) indicators, substantially lower than the other administrative levels. Prefecture laboratories scored weak on ‘Qualification and Accreditation’ (59%), but interestingly scored higher than provincial laboratories for ‘External Communication’ and ‘Budget’.

#### Documents

County-level laboratories scored lower than prefecture- and provincial-level laboratories for each of the three indicators that comprise the ‘Documents’ module (Fig. 2). Although county-level laboratories scored good on ‘Biosafety Documents’, the overall weak module ranking was due to weak scores for ‘Document Control System’ (65%) and ‘Quality Procedures’ (65%).

#### Laboratory testing performance

Figure 3 shows the variability across the three administrative levels for each of the four Laboratory Testing Performance indicator scores. The overall weak module score for prefecture level laboratories was due to weak scores in Virology (62%) and Parasitology (50%); however, these same laboratories earned strong scores for the ‘Food Testing’ indicator. Interestingly, county-level laboratories scored higher for all four indicators than their provincial and prefecture counterparts.

#### Facilities

The ‘Facilities’ module scores reflect the average across two indicators (Fig. 4.). As with the ‘Documents’ module, county-level laboratories scored < 70% for these indicators, lower than both prefecture and provincial laboratories.

#### Biorisk management

Similar to the ‘Documents’ module, county-level laboratories scored lower than prefecture- and provincial-level laboratories for each of the three indicators that comprised the ‘Biorisk Management’ module. Laboratories at all levels scored strong or good on ‘Implementation and Operation’ indicator, while scores are weak on ‘Biorisk Assessment and Control’ indicator.

#### Public health functions

Performance on the ‘Public Health Functions’ module was considered weak or very weak across the three administrative levels. This finding reflects low scores for each of the three ‘Public Health Function’ indicators (Fig. 6). Prefecture- and county-level laboratories performed very weak in ‘Surveillance and Response’ and ‘Public Health Reporting’ (40 and 30%, respectively). Provincial laboratories scored very weak (45%) in ‘Specimens for Public Health Purpose’.

### Module performance for public health and clinical laboratories

Hospital-based clinical laboratories and public health laboratories generally received similar scores for seven of the 11 modules included in this assessment (Table 2). Differences were observed for ‘Organization and Management’, ‘Facilities’, and ‘Biorisk Management’ modules where hospital-based clinical laboratories scored higher than their public health counterparts at each administrative level. At the same time, CLs were substantially weaker than PHLs in ‘Public Health Functions’, greatly impacting the overall score for this module, particularly at the provincial and prefecture levels. Most noteworthy are the lower scores among CLs for ‘Public Health Functions’. We have included figures comparing these module indicators between public health and clinical laboratories in the Additional files 1, 2, 3 and 4.

## Discussion

According to this quantitative laboratory assessment in multiple provinces of China on laboratory capacity as required by IHR, laboratories in Guangxi and Guizhou Provinces are generally performing well for most of the LAT modules. Two modules, ‘Specimen Collection, Handling and Transport’ and ‘Equipment’ received strong scores and six other modules received good scores. These findings likely reflect the long-term commitment of national and local Chinese leaders for laboratory capacity and system development. However, our assessment also identified a number of substantial performance gaps, particularly for the module ‘Public Health Functions’, where clinical laboratories were particularly weak in surveillance and response capacities. Additionally, county-level laboratories generally scored lower than prefecture and provincial laboratories on a number of modules.

The weak or very weak performance scores for ‘Public Health Functions’ were consistent across provincial-, prefecture-, and county-level laboratories and lagged far behind the other 10 capacities required by IHR. These scores were primarily driven by low surveillance and response capacity among clinical laboratories. Hospital-based CLs play an essential role in fulfilling public health functions, particularly for specimen collection, clinical diagnostics and specimen referral, as well as reporting diagnostic test results in an accurate and timely manner. Improving collaboration between laboratories across the two sectors and sharing testing and confirmatory test results could strengthen both laboratories, especially when confronted with emerging and re-emerging diseases. Since CLs scored high on ‘Biorisk Management’ [10] at all administrative levels, such collaboration could also reinforce implementation of bio-risk control measures among PHLs through cross-sector trainings and meetings.

Under the guidance of the Law of Infectious Diseases Prevention and Treatment [11], the National Health and Family Planning Commission (formerly called Ministry of Health) in China currently supports a number of disease-specific surveillance systems that cover field investigations, diagnostics, and reporting for individual etiologies [12]. Inadvertently, this vertical approach for many different diseases and syndromes could have negatively impacted capacity for implementing public health functions in both PHLs and CLs [12]. This is likely most pronounced in provinces with limited laboratory resources. Integrating disease-specific systems into surveillance and laboratory networks could help maximize available resources as well as strengthen engagement of all laboratories in surveillance and response activities.

The use of a quantitative scoring system allowed us to identify critical differences in laboratory capacity for a number of modules across provincial, prefecture, and county-level laboratories. The most noteworthy differences were observed for ‘Organization and Management’, ‘Documents’, ‘Facilities’, ‘Laboratory testing’ and ‘Biorisk Management’. With the exception of ‘Laboratory Testing’, performance scores for each of these modules were typically highest for provincial laboratories, followed by prefecture laboratories and lowest for county-level laboratories. This finding may be partially explained by financial constraints often experienced by county-level laboratories [13]. These constraints can negatively affect staff recruitment, personnel training and capacity development, as reflected in the weak county-level scores for ‘Facilities’ and ‘Human Resources’.

Since county- as well as prefecture-level laboratories are responsible for field-based epidemiological investigations and perform initial specimen collection and testing, the weak scores for these modules need to be addressed. Available tools such as the IHR implementation roadmap [14] to guide laboratory management and quality improvement could be used to address gaps across all levels. For instance, we can match an assessed capacity, such as ‘Documents’, with the corresponding phase of the roadmap and implement interventions to guide improvements in this capacity area. Interventions can include developing and improving procedures for testing, increasing availability and quality of laboratory equipment, strengthening inventory management as well as biosafety practices. Checklists can be used to verify completion of each activity. Additionally, these tools, along with support from provincial laboratories, could help prefecture laboratories meet accreditation requirements and competences [1].

Laboratories included in this assessment scored an average of 71% for the module ‘Laboratory Testing Performance’. National guidance was published in 2004 describing the categories of tests that PHLs at each administrative level should be able to detect [15]. This guidance includes testing for diseases caused by bacteria, viruses, parasites and *Rickettsia.* It also includes testing conducted in preventive and check-up settings, such as for non-communicable diseases as well as chemicals and hazards in water, cosmetics, and the environment. According to the guidance document, PHLs at the provincial, prefecture, and county levels should be able to perform at least 433, 359 and 179 pathogen-specific diagnostic tests, respectively. Our assessment scored capacity according to this guidance and assessed 82 categories of tests for each administrative level, which is approximately one-fifth, one-fourth, and one-half of the required categories of tests for provincial, prefectural and county levels. Additionally, we did not compare testing types or accuracy for pathogen detection. This may partially explain the higher scores for county-level laboratories when compared to the provincial and prefecture laboratories included in this assessment.

Based on our assessment, national guidance appears to be generally helpful for PHLs. A similar guidance document addressing the specific challenges for CLs along with a training component aimed at increasing capacity in Public Health Functions could be beneficial. Such guidance and training will require closer collaboration and long-term commitments by the agencies managing the separate laboratory sectors.

We collaborated with international, national, and local public health staff and hospital administrators to design and conduct this laboratory assessment. This collaborative approach increased the likelihood that the assessment would be successfully implemented at each administrative level and across the two sectors. We were able to customize the LAT to the China laboratory structure; however, the resulting data may not applicable or comparable to other types of laboratories or to other countries. The tool allowed us to use the same quantitative score system while modifying the cut-offs for strong, good, and weak as laboratory capacity improves over time. WHO recently launched a new IHR monitoring and evaluation framework, including the Joint External Evaluation (JEE) tool [16]. This framework will combine self-evaluation, peer review and voluntary external evaluations for assessing IHR laboratory core capacities. Prior to future assessments, the LAT may need to be aligned with this new WHO IHR monitoring and evaluation framework.

We collected self-reported data from laboratory managers, and several different team members conducted the field verification and inspection visits for all modules to minimize possible bias. Project leads trained staff at each participating laboratory on the use of the self-assessment tool as well as members of the field visit teams prior to data collection. Nevertheless, PHL and CL staff could have had different interpretations of the survey questions, such as on biorisk management, due to their work focus. Additionally, we elected to use a multi-stage sampling approach that reduced travel time and resources required to complete the assessment (i.e., visiting county level laboratories located in only two different prefectures in each Province). Consequently, our findings, particularly at county-level, may not be representative for other laboratories in the two provinces. According to our local partners differences between prefectures are likely to be minimal.

## Conclusion

We were able to successfully implement a quantitative assessment of laboratory capacity in two resource-limited provinces in China. From this assessment, laboratories are generally performing well in laboratory capacity as required by IHR, with the exception of ‘Public Health Functions. Addressing gaps identified in this module, particularly in surveillance and response capacities, are likely to have the greatest positive impact for global health security and IHR requirements. We also recommend targeted interventions for county-level laboratories, where capacity was weak for a number of modules. Opportunities to strengthen collaboration between public health and clinical sectors could also be beneficial. Findings from this assessment can serve as a baseline to evaluate the impact of these targeted interventions.

## Additional files

Additional file 1: Performance scores combined across three administrative levels (county, prefecture and province) for the 11 assessment modules for the 28 laboratories by laboratory type (public health and clinical), Guangxi and Guizhou Provinces, China, 2014. (DOC 95 kb)

Additional file 2: Provincial-level performance scores for the 11 assessment modules for the 28 laboratories by laboratory type (public health and clinical), Guangxi and Guizhou Provinces, China, 2014. (DOC 93 kb)

Additional file 3: Prefecture-level performance scores for the 11 assessment modules for the 28 laboratories by laboratory type (public health and clinical), Guangxi and Guizhou Provinces, China 2014. (DOC 90 kb)

Additional file 4: County-level performance scores for the 11 assessment modules for the 28 laboratories by laboratory type (public health and clinical), Guangxi and Guizhou Provinces, China 2014. (DOC 89 kb)

## Abbreviations

CDC: Centers for Disease Control and Prevention
CLs: Clinical laboratories
IHR: International Health Regulations
LAT: Laboratory Assessment Tool
PHLs: Public health laboratories
WHO: World Health Organization
