# Supplementary figures and images for: Capacity assessment of the health laboratory system in two resource-limited provinces in China
Source: BMC Public Health. 2019 May 10;19(Suppl 3):467. doi: 10.1186/s12889-019-6777-2 (PMC6696693; doi:10.1186/s12889-019-6777-2)

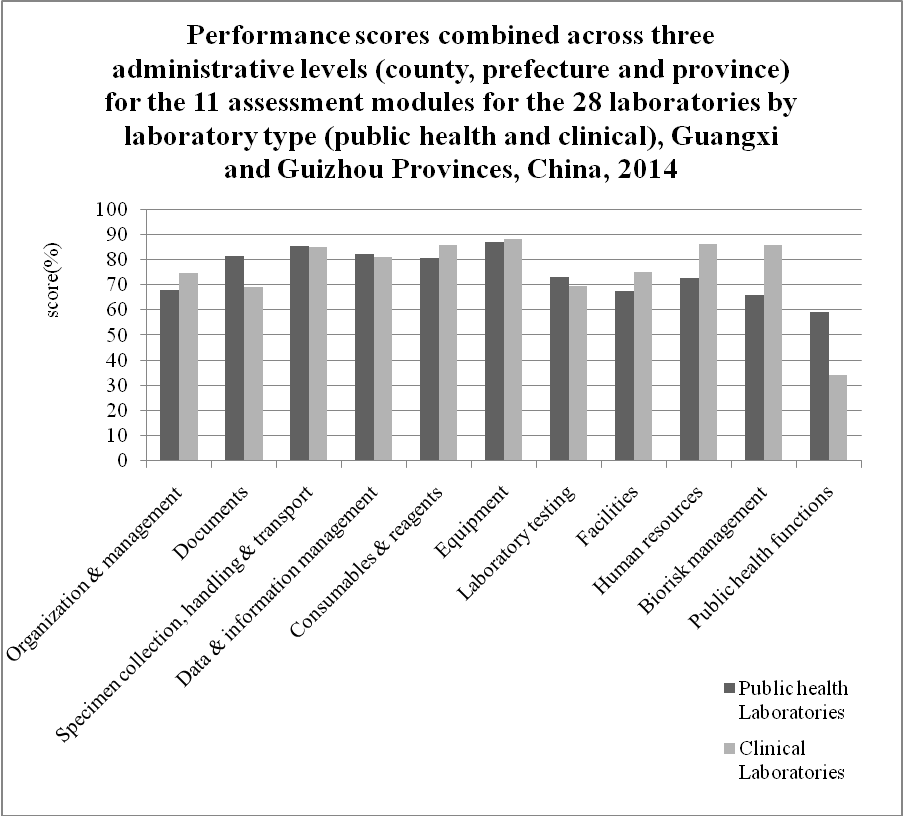

Supplement: Supplementary file 1 — Performance scores combined across three administrative levels (county, prefecture and province) for the 11 assessment modules for the 28 laboratories by laboratory type (public health and clinical), Guangxi and Guizhou Provinces, China, 2014. (DOC 95 kb) [file 12889_2019_6777_MOESM1_ESM.doc]

**
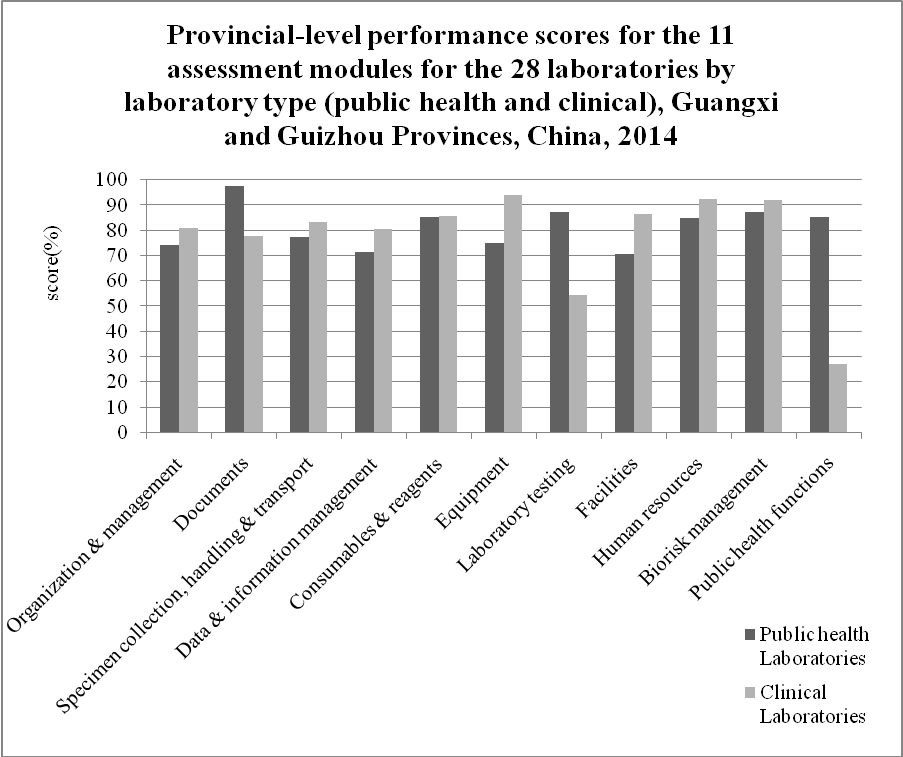
**

Supplement: Supplementary file 2 — Provincial-level performance scores for the 11 assessment modules for the 28 laboratories by laboratory type (public health and clinical), Guangxi and Guizhou Provinces, China, 2014. (DOC 93 kb) [file 12889_2019_6777_MOESM2_ESM.doc]

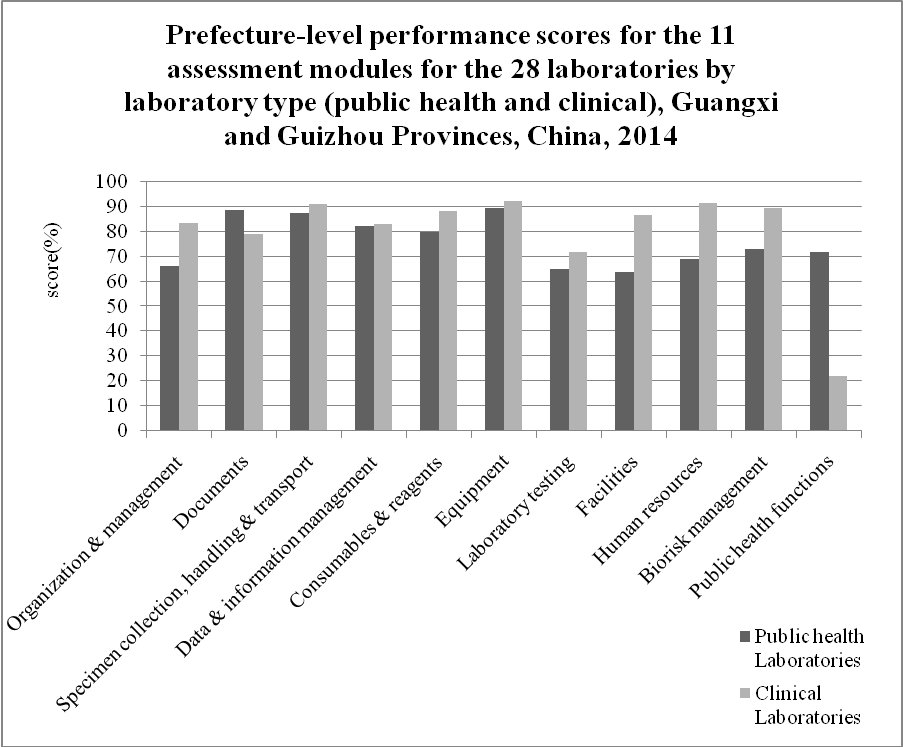

Supplement: Supplementary file 3 — Prefecture-level performance scores for the 11 assessment modules for the 28 laboratories by laboratory type (public health and clinical), Guangxi and Guizhou Provinces, China 2014. (DOC 90 kb) [file 12889_2019_6777_MOESM3_ESM.doc]

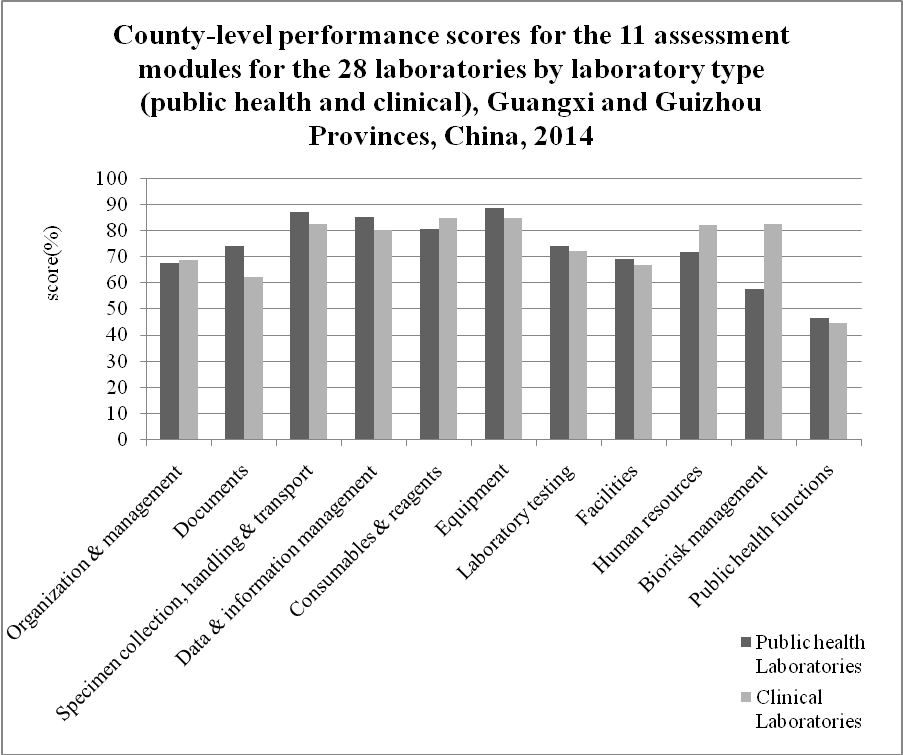

Supplement: Supplementary file 4 — County-level performance scores for the 11 assessment modules for the 28 laboratories by laboratory type (public health and clinical), Guangxi and Guizhou Provinces, China 2014. (DOC 89 kb) [file 12889_2019_6777_MOESM4_ESM.doc]
